# Morphological Properties of Mass–Spring Networks for Optimal Locomotion Learning

**DOI:** 10.3389/fnbot.2017.00016

**Published:** 2017-03-27

**Authors:** Gabriel Urbain, Jonas Degrave, Benonie Carette, Joni Dambre, Francis Wyffels

**Affiliations:** ^1^IDLab, Electronics and Information Systems Department, Ghent University – imec, Ghent, Belgium

**Keywords:** morphological computation, mass–spring networks, morphological control, physical reservoir computing, soft robotics

## Abstract

Robots have proven very useful in automating industrial processes. Their rigid components and powerful actuators, however, render them unsafe or unfit to work in normal human environments such as schools or hospitals. Robots made of compliant, softer materials may offer a valid alternative. Yet, the dynamics of these compliant robots are much more complicated compared to normal rigid robots of which all components can be accurately controlled. It is often claimed that, by using the concept of morphological computation, the dynamical complexity can become a strength. On the one hand, the use of flexible materials can lead to higher power efficiency and more fluent and robust motions. On the other hand, using embodiment in a closed-loop controller, part of the control task itself can be outsourced to the body dynamics. This can significantly simplify the additional resources required for locomotion control. To this goal, a first step consists in an exploration of the trade-offs between morphology, efficiency of locomotion, and the ability of a mechanical body to serve as a computational resource. In this work, we use a detailed dynamical model of a Mass–Spring–Damper (MSD) network to study these trade-offs. We first investigate the influence of the network size and compliance on locomotion quality and energy efficiency by optimizing an external open-loop controller using evolutionary algorithms. We find that larger networks can lead to more stable gaits and that the system’s optimal compliance to maximize the traveled distance is directly linked to the desired frequency of locomotion. In the last set of experiments, the suitability of MSD bodies for being used in a closed loop is also investigated. Since maximally efficient actuator signals are clearly related to the natural body dynamics, in a sense, the body is tailored for the task of contributing to its own control. Using the same simulation platform, we therefore study how the network states can be successfully used to create a feedback signal and how its accuracy is linked to the body size.

## 1 Introduction

Since its very early formulation, control theory has tried to automate increasingly complex systems (Fernández Cara and Zuazua Iriondo, 2003). The first implementations of PID controllers using feedback to regulate non-linear systems only originated in the first part of the twentieth century and were improved considerably, in particular with the progress in aerospace. More recently, with the evolution of the computation power and the advances in machine learning, the focus has evolved toward the control of highly compliant systems with many degrees of freedom. Passive compliant robots indeed possess dynamical properties closer to animal bodies, whose performances can still not be reached, and show a real advantage for solving complex tasks in noisy human environments.

However, the framework for a theory allowing a deep understanding of such control systems—and hence engineering opportunities—is still under construction. It is largely believed that the concept of morphological computation can partly answer this issue, as it enables more fluent and robust motion control while providing adapted embodied controllers that use the body itself as a computational mean (Paul, 2006; Pfeifer and Bongard, 2006).

Nonetheless, the concept of morphological computation does not have a clear definition as discussed in Müller and Hoffmann (2016). In Füchslin et al. (2013), the authors refer to the first International Conference on Morphological Computation in Venice in 2007, where it was defined as “any process that serves a computational purpose, has clearly assignable input and output states, is programmable (i.e., the behavior can be adapted by varying a set of parameters) and has a sort of teleological embedding.” This definition is however rather broad as it also includes every traditional digital computing means. Hereafter, we will restrict our definition to any way of increasing efficiency of computation or control in terms of energy, memory, time, etc. by outsourcing computational tasks to analogical physical systems. This interpretation follows the work of Pfeifer and Bongard (2006) where morphological computation refers to “certain processes are performed by the body that otherwise would have to be performed by the brain” or with the experiments conducted in Hauser et al. (2011). Moreover, it constitutes a fundamental motivation to embodiment which states that steps toward adaptive intelligence do not only come from the controller complexity but also from the interactions with the body and the environment. Broader analysis about the quantification of morphological computation as well as the trade-offs with informational computation include Polani (2011), Zahedi and Ay (2013), Haeufle et al. (2014), Hoffmann and Müller (2014), and Ghazi-Zahedi et al. (2016).

Illustrative applications of morphological computation and embodiment for locomotion are numerous in biology and robotics. For instance, Dickinson et al. (2000) provide an analysis of how animals succeed in efficient locomotion using their muscles not solely as motors but to provide multiple functions varying from brakes to springs and struts. The *passive walker* in McGeer (1990) constitutes an extreme example of an engineered robot exploiting the same concept. This two-legged physical structure is able to walk down a slope in a very natural way without any actuation. This work has been extended later in Collins et al. (2005) to robots with low-power actuators. They show a walking pattern that looks natural and energy efficient compared to traditional stiff controlled robots. In other fields of robotics, we can also cite the works of Iida and Pfeifer (2006) or Degrave et al. (2015), in which dynamical properties of compliant quadruped robots are used to provide low power consumption, to reduce controller computational complexity, and to observe natural transitions between gaits. Examples that clearly benefit from compliance to improve moving can also be found, among others, in Cham et al. (2004) which focuses on hexapod locomotion.

A practical implementation of morphological computation can be inspired from Reservoir Computing (RC). RC denotes a computational framework that enables the approximation of a broad range of dynamical behaviors for which a precise model is not available. RC originates from the domain of recurrent neural networks and is mainly based on the theories of Echo State Networks (ESN) and Liquid State Machines (LSM) as outlined in Lukoševičius and Jaeger (2009). At the time of their introduction, they offered a solution to the training of Recurrent Neural Networks (RNN), which was still considered difficult. They avoided having to train feedback connections and the problems with bifurcations this brings, i.e., the discontinuities in the network outputs observed for some points in the parameter space, by training only the synaptic connections of the readout nodes. The core architecture consists of a randomly connected RNN, the *reservoir*, for which the synaptic weights are sampled from some distribution and then globally rescaled to tune the dynamical regime close to the edge of chaos. RC also resulted in different robotics applications as learning of inverse kinematics of an iCub robot arm from a neural reservoir in Reinhart and Steil (2009) or the creation Central Pattern Generators (CPG) to control human movements in Wyffels et al. (2014) and hexapod locomotion in Dasgupta et al. (2015).

As the reservoir network is constituted of randomly connected non-linear entities, many physical dynamical systems presenting sufficiently complex transformations of their inputs provide similar dynamical properties and can be used as reservoirs. For instance, it has been demonstrated in Hauser et al. (2012) that generic types of physical bodies such as Mass–Spring–Damper (MSD) networks are able to approximate any given time-invariant filter with fading memory and generate adaptive periodic patterns autonomously when a feedback loop is added. This extension of RC is generally referred to as Physical Reservoir Computing (PRC). The expensive step of computing the reservoir transformation is now outsourced to a physical system’s natural dynamics. This means that the neuron states will not be explicitly updated digitally anymore, but this computation is transferred to the body’s dynamical evolution. Only the readout layer only needs to be engineered, most often using digital computing.

The main advantage of PRC lies in the parallelism of the computations in the physical reservoir and, in the case of robotic locomotion, in the fact that the transformations computed by the robot body are a natural result of the gait. However, PRC is essentially a supervised machine learning technique. By contrast, robotic control is intrinsically a reinforcement learning problem, in which the optimal desired actuator signals are not known *a priori*. In addition, successful reservoir implementations often require the observation of the reservoir state at many different points. In robotics, this implies that for each observation point a sensor needs to be installed.

Numerous applications of PRC have been demonstrated in the past decade. In robotics, highly compliant robot models have been addressed for example to MSD networks in Hauser et al. (2011) (simulation only), tensegrity structures in Caluwaerts et al. (2014) or a real soft robotic platform inspired by an octopus arm in Nakajima et al. (2014, 2015). Closed-loop control of quadruped robot exploiting a spine made with soft material as a reservoir can be found in Zhao et al. (2013). Simulations or implementations of PRC outside robotics include water ripples in Fernando and Sojakka (2003), electro-optical devices in Larger et al. (2012), or pure optical devices in Brunner et al. (2013) and Vandoorne et al. (2014).

This paper presents two main research objectives. First, we design a small scalable simulation setup to provide empirical compliance studies on the locomotion of MSD networks. To our knowledge, such an analysis does not yet exist and should help to evaluate the potential of compliance for locomotion in terms of robustness, efficiency, and stability. To this end, three main experiments are conducted. The first experiment gives an overview about how increasing the number of nodes in a MSD network leads to more stable locomotion. The second experiment provides an analysis on the optimal frequency range for the setup, and the third experiment explores the maximal reachable speeds for different driving powers and underlines the limitations of the design to get high performance. In the second part, we analyze the computational capacity of a MSD body to generate motor control signals and integrate them as a regulation feedback to a forward controller.

## 2 Open-Loop Control

### 2.1 Materials and Methods

To run our experiments and analysis, we designed a MSD network simulator directly implementing mechanical equations using *Python* and *Numpy*.^1^ These networks, inspired by Hermans et al. (2014) and Caluwaerts et al. (2013), consist of a set of nodes with mass, connected by spring–damper links which are all actuated separately. The simulation can be performed either in 2D or 3D.

#### 2.1.1 Mass Spring Networks

**Figure 1:**
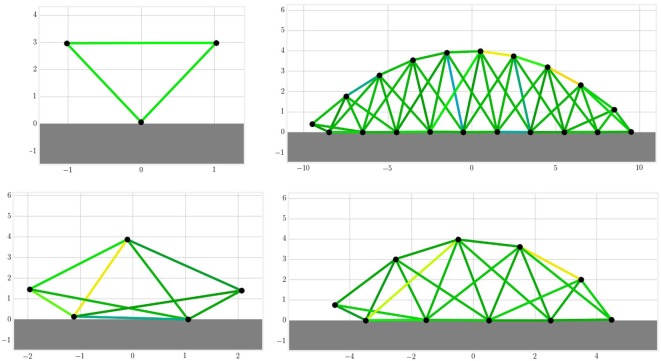
**The MSD structures are build automatically with a simple morphology that takes the number of nodes and connections as an input**. On the figure above individuals with three, five, ten, and twenty nodes are drawn in a 2D space. Each black circle represents a mass and each line a set of spring and damper in parallel. The colors indicate the current amplitude of actuation.

The MSD morphology is presented in Figure 1. Each of the *N* nodes, except those at the end or beginning, is sparsely connected to its closer neighbors by *C* connections. The total number of springs in the network *S* can be easily deduced using geometry:

(1) $$S=\left(N-1-\frac{C∕2-1}{2}\right).\frac{C}{2}.$$

Each node *i* ∈ {1, … , *N*} is represented by its mass *m_i_*, whereas the passive parameters for each connection are the spring stiffness *k_j_* and the damper coefficients *d_j_* for *j* ∈ {1, … , *S*}. In this paper, the notion of compliance will be used. It is defined as the inverse of the stiffness 1/*k_j_*. If not specified, the default values used in the following experiments are *N* = 20, *C* = 3, *m_i_* = 1 kg, *k_j_* = 100 N/m, and *d_j_* = 10 Ns/m.

In our model, the acceleration, speed, and position of each mass are updated using the force vector **F***_i_* which combines the gravity force, the spring force, the damping force, and the air friction force:

(2) $${\text{F}}_{i}={\text{F}}_{i}^{s}+{\text{F}}_{i}^{d}+{\text{F}}_{i}^{g}+{\text{F}}_{i}^{a},$$

where

- ${\text{F}}_{i}^{s}$ is the spring force vector applied on the node *i* and equals the sum of the *j* ∈ {1, … , *C*} connected non-linear springs forces for which the equations can be found in Palm (1999):
  (3) $${\text{F}}_{j}^{s}=-k_{j}.\frac{{\text{l}}_{j}}{l_{j}}.\left(\left(l_{j}-l_{j,0}\right)+\frac{\alpha }{l_{j,0}^{2}}.{\left(l_{j}-l_{j,0}\right)}^{3}\right).$$
  In this equation, **l***_j_* represents the spring length vector and *l_j_*_,0_ its reference length. The variable α is a non-linearity coefficient which will induce a saturation of the spring force for large extension lengths. It also takes inspiration from the work of Hauser et al. (2011) which demonstrates the importance of these non-linearities from a computational consideration.
- ${\text{F}}_{i}^{d}$ is the damper force vector applied on the node *i* and equals the sum of the *j* ∈ {1, … , *C*} connected dampers:
  (4) $${\text{F}}_{j}^{d}=d_{j}\;.\;\frac{{\text{v}}_{j}}{v_{j}}\;.\;\left(v_{j}-v_{j,0}\right),$$
  where **v***_j_* is the vector of extension speed.
- ${\text{F}}_{i}^{g}$ is the gravity force vector:
  (5) $${\text{F}}_{j}^{g}=g\;.m_{i}.\;{\text{x}}_{i}^{y},$$
  where *g* is the gravity constant and equals 9.81 m/s^2^.
- ${\text{F}}_{i}^{a}$ represents the drag force induced by air friction. It is assumed proportional to the speed:
  (6) $${\text{F}}_{j}^{s}=-a\;.\;{\text{v}}_{i},$$
  where *a* is the coefficient of air friction and equals 0.1 Ns/m. It has been included to avoid unrealistic models with very high speed. However, these did not occur in the experiments presented in this paper.

The ground reactions are modeled by setting the vertical velocity to zero and the horizontal friction coefficient to infinite. The masses perfectly stick to the ground as soon as they touch it. This is a hard constraint that can impact the nature and the performance of locomotion. However, it simplifies the study of the body influence by assessing perfect friction conditions in every simulation.

#### 2.1.2 Control

To actuate the spring using a control signal, we modulate the reference lengths of the springs *l_j_*_,0_. In the simplest and default case, this will be represented by a simple sinusoidal signal like in Hermans et al. (2014):

(7) $$l_{j}\left(t\right)=l_{j,0}\;.\;\left(1+\;a_{j}\;.\;\text{sin}\left(\omega_{j}\;.\;t+\phi_{j}\right)\right).$$

It induces a set of tunable parameters *l_j_*_,0_, *ω^j^*, *ϕ^j^* for each spring in the simulation.

#### 2.1.3 Physics Solver

The simulation time is discretized using *K* time steps *t_k_*, and equations are solved numerically using the Verlet algorithm as described in Thijssen (2007). The Verlet integrator leads to more accurate trajectories, especially for periodic oscillations where energy is rigorously conserved due to the time reversibility of this operator. For non-periodic trajectories, one can prove that due to symplecticity, the energy does not drift away and errors remain bounded as demonstrated in Yoshida (1990). Although it is more accurate, the Fourth-Order Runge–Kutta integrator requires four force evaluations per update step and is not symplectic. In our implementation, the update equations are slightly changed in order to take the effect of the ground reactions into account.

#### 2.1.4 Loss Function

The goal is to develop a generic approach to obtain robust locomotion in open loop without prior knowledge about the body dynamics. In the case of simulated MSD networks, this implies the optimization of controller and morphology parameters for each specific network. This can be formulated as

(8) $$\hat{\theta }=\text{arg }\underset{\theta }{\text{max}}\;f\left(\theta \right).$$

where the score function *f*(***θ***) and the optimization algorithms are detailed below. Typically, the optimized parameters ***θ*** of the MSD network are the controller amplitude *a_j_* between 0 and 0.25, its frequency between 0 and 10 Hz, its phase *ϕ_j_* between 0 and 2π, and the spring stiffness *k_j_* between 0 and 100 N/m. To synchronize the actuators together and impose the fundamental frequency, the angular speeds *ω_j_* are fixed to the same value. In the case of a MSD with *N* = 20 nodes connected to their six closest neighbors (*C* = 6), this represents a total number of springs *S* = 54 (see equation (1)) and therefore 163 parameters to optimize. Locomotion characterization and evaluation is performed through two performance metrics:

- **Distance traveled** *D*: the difference between the centers of mass at the end and at the beginning of the simulation. This function is determined by the full locomotion sequence along the simulation.
- **Power efficiency** *P*: the power dissipation of the non-linear spring actuators can be approximated according to Palm (1999):
  (9) $$P=\sum_{j}\;k_{j}\;.\;\frac{a_{j}^{2}\;\;l_{j,0}^{2}\;\left(1+\alpha^{2}a_{j}^{2}\right)}{4\pi },$$
  in which *a_j_* are the relative amplitudes, α is the spring non-linearity factor, and *l_j_*_,0_ are the reference lengths of the springs.

**Figure 2:**
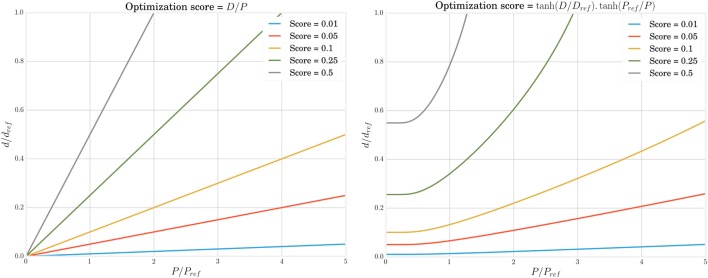
**The efficiency score to quantify locomotion quality increases with traveled distance and decreases with dissipated power**. However, taking directly the ratio between both metrics (left) could lead to optima close to the origin, i.e., where the body barely moves. Using a hyperbolic tangent (left) solves this problem for small powers but requires to select *P_ref_* and *D_ref_* carefully to avoid a saturation due to the measure itself.

Using the ratio of distance to power is unsatisfactory, as this could result in robots that consume very little power because they barely locomote. Instead, we will use the following power efficiency score displayed in Figure 2:

(10) $$f\left(\theta \right)=\text{tanh}\;\left(\frac{D\left(\theta \right)}{D_{\mathrm{ref}}}\right)\;\;.\;\text{tanh}\;\left(\frac{P_{\mathrm{ref}}}{P\left(\theta \right)}\right),$$

in which *D_ref_* and *P_ref_* are reference values allowing to normalize and homogenize the scores. As it is desirable to operate in the linear regime and avoid saturation of the score, we set them to 3,600 and 100, respectively, following the statistics of the observed distance and power values.

#### 2.1.5 Optimization

The aim is to develop an optimization approach that can be applied to highly compliant physical robots, without any need for an analytical model for the body dynamics. The Covariance Matrix Adaptation Evolution Strategy (CMA-ES) as formulated in Hansen (2006) has been selected from a pool of different optimization methods. Indeed, it fits very well for browsing non-convex parameter landscapes with a lot of local minima. In addition, it presents a good convergence speed and requires very few initialization parameters:

- The initial parameter distribution is a Gaussian centered in 0.5 and with a SD of 0.2 after normalization of all parameters.
- The population size, the step size, and covariance matrix parameter are set to their default values as recommended in Hansen (2006).
- The iteration number is set to ensure convergence, which will be assessed qualitatively by observing saturation in the score evolution.

### 2.2 Results

In this section, we assess the influence of the MSD network size and compliance on the best locomotion speed found, on the power consumption, and on the noise robustness in our specific example. Three different experiments are described in this context. In the first one, we increase the number of mass nodes in the network to determine its influence on locomotion efficiency. The second investigates how optimal compliance is related to the morphology parameters and the locomotion frequency. Finally, we discuss how the optimized gait changes when the driving power is constrained.

#### 2.2.1 Morphology Analysis

The choices made during the design of a system can contribute to more efficient and robust behaviors for solving sophisticated tasks. In the case of the MSD setup, we can intuitively assume that increasing the number of nodes will broaden the space of available trajectories, therefore increasing the number of optima at the expense of a longer learning process. It is interesting to note that such a tuning does not necessarily imply an increase of complexity, in the sense of the definition presented in Lungarella and Sporns (2006).

To verify this assumption, we have optimized open-loop locomotion controllers for networks with increasing number of nodes and springs. As mentioned before, this optimization consists in tuning the actuators’ amplitudes and phases, the spring constants, and the global frequency of locomotion. Other parameters of the MSD network are set to the same value for all bodies, except for the nodes mass. This is normalized by the number of nodes, such that the total mass of the MSD network (20 kg) remains the same in every simulation and the power levels required for locomotion can be compared.

In order to converge toward stable gaits, we add random acceleration impulses during the simulation. Their value is centered around 10% of the mean absolute acceleration and applied on random nodes 5% of the time. In the CMA-ES algorithm, the number of iterations is tuned specifically for each optimization to ensure convergence, since optimizing small structures will converge faster than larger ones. From each optimization run, the best individual is retained. Each optimization is repeated five times in order to average the results and obtain an estimate of the variability of our observations.

**Figure 3:**
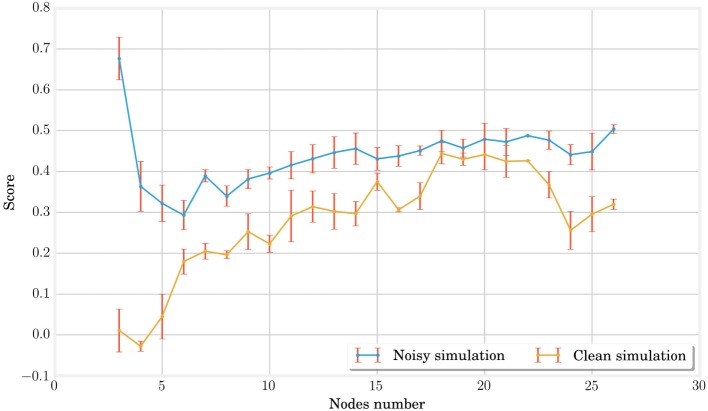
**In this graph, the best individuals for CMA-ES optimizations of different MSD networks are plotted in blue**. Other simulations without noise are then performed on the same individuals with the same parameters in order to identify the outliers due to noise and qualify the stability of locomotion. The low performance of 3, 4, and 5 nodes MSD structures indicate unstable gaits. For larger structures, the score first increases with the number of nodes but saturates rapidly for networks of more than twenty nodes.

**Figure 4:**
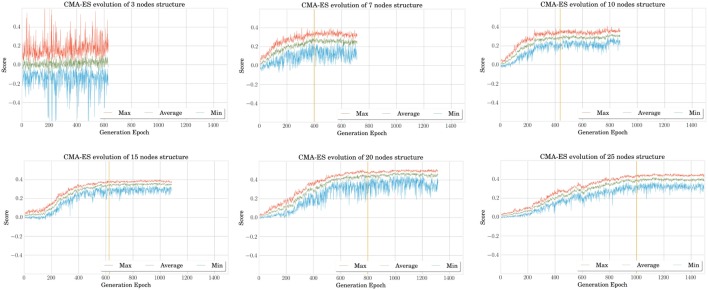
**In this graph representing the CMA-ES evolution for different structures, we can qualitatively observe that the convergence time, whose estimation is given by the vertical yellow lines, increases with the MSD network size**. This is expected as the problem becomes more complex and the number of optimized parameters is higher as well. When the structure is too simple such as the three nodes (one in the upper left corner), the problem cannot converge and the best results encountered during the exploration are mainly due to the random noise added in simulation.

Figure 3 shows the evolution of the averaged best individual score for increasing body size in blue. From left to right, we observe that the scores rapidly decrease for structures of up to five nodes before steadily increasing again. However, the good results in the first part of the curve should be interpreted carefully, taking their robustness to noise into account. To assess this property, we also represented the scores obtained for simulation using the same parameters but without noise on the same figure. We notice that this difference decreases with the number of nodes. This shows that structures with more nodes are more robust to the noise added during the simulation. The evolution of the CMA-ES algorithm represented in Figure 4 also supports this hypothesis. It shows that the optima of the structures with a small number of nodes are found randomly instead of through convergence of the algorithm, unlike the structures with more nodes. High scores originate from these bodies’ reduced stability. This makes them very sensitive to impulse noise as small disturbances can either make them fall over or push them forward. They can therefore rightly be regarded as outliers.

**Figure 5:**
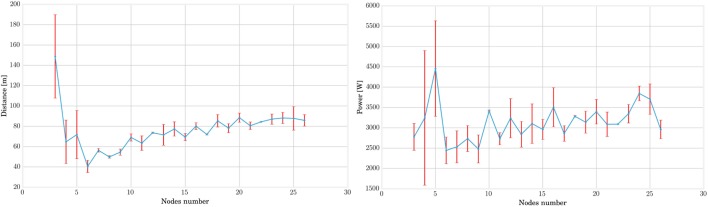
**By displaying separately the distance and power components in the loss function of the CMA-ES optimization, we can acknowledge that the observed variation for different nodes number are mainly due to the distance**. As expected by the normalization factor, the driving power remains sensibly equals for each structure.

It is finally interesting to note that the score increases gradually starting from six nodes but quickly saturates. A more detailed analysis in Figure 5 shows that this is due to better performances in terms of traveled distance, whereas dissipated powers are very similar. However, note that this is achieved at the expense of a longer learning process, as pointed out by the number of epochs represented on the graphs X axis of Figure 4.

In conclusion, this experiment points out that increasing the number of nodes and springs in the MSD networks leads to an increased robustness to external noise and better speed performances.

#### 2.2.2 Frequency Range Analysis

In this second set of experiments, we try to evaluate the nature of a link between robot compliance, which is defined by 1/*k*, the inverse of spring stiffness, and the optimum efficiency of locomotion.

The resonance frequency of a MSD system with one unique node and spring equals $\sqrt{k∕m}$. It ranges from 0.6 to 1.8 Hz for the *m_i_*, *k_j_*, and *d_j_* values that we are using in our setup (as a reminder, *m_i_* varies with the number of nodes). There is therefore a bijective function between compliance and resonance frequency. By extension, we can formulate the hypothesis that the resonance frequency of a MSD structure is directly coupled to its compliance. Since they are composed of several masses and springs, we can expect that the bandwidth of the resonance peak will broaden but still appear at the same frequency.

With this assumption, the study of correlation between compliance and locomotion efficiency can be reformulated to focus on the link between actuation frequency and efficiency. Previous work such as Buchli et al. (2006) for robotic systems or McMahon and Cheng (1990) for models of mammalian gaits highlighted such a link: self-learning systems with different morphology properties tuned their actuation frequency to the resonance of the structure to reach optimal performance in locomotion.

**Figure 6:**
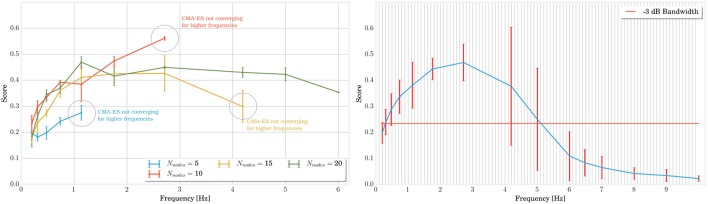
**The two graphs in this figure show the evolution of score performances with the fundamental frequency of locomotion**. On the left side, we separated the result according to the number of nodes in the structure. By applying a simple CMA-ES convergence assertion, we notice that the operating range can extend to higher frequency for larger structures. On the right side, we combined all structures to determine a −3 dB bandwidth ranging from 0.3 to 5.2 Hz.

In this setup, MSD structures with 5, 10, 15, and 20 nodes were optimized several times by fixing their global frequency to values between 0 and 10 Hz. In Figure 6 (on the left), we have represented the results for different numbers of nodes. Each optimization corresponds then to a point on the graph. For some of those points, however, the optimization process was not able to converge to a gait that is both stable (whose pattern does not change in time) and robust (allowing external noisy perturbations). In the graph, this failure is particularly true for structures with few number of nodes simulated at high frequencies. A first empirical conclusion is that the robustness of MSD networks at high frequencies increases with the number of nodes. This represents an additional advantage concerning the size of the system along with the discussion from previous section. In terms of score, however, there is no significant difference between the topologies, and their optimal bandwidths are very similar. The optimal scores are a little lower only for the 5 nodes structures, which corroborates the results from the previous experiment. To get a more accurate measure of the bandwidth, it may even be interesting to combine all the results as they possess very similar resonance frequency. This is presented on the right side of Figure 6 where we can observe that the structure is optimal over a 3 dB bandwidth in the range [0.3; 5.2 Hz]. The large confidence intervals around 4 and 5 Hz are again explained by the absence of convergence for the structures with a low number of nodes.

To sum up, this experiment provides guidance on the choice of compliance values in the design of a MSD network for locomotion. Choosing the global compliance to optimize a robot of a given mass is conditioned by the frequency at which we plan to actuate the robot. Also, structures with more nodes tolerate a broader range of frequencies while keeping stability.

#### 2.2.3 Performance Limits with Constrained Power

So far, we have used a loss function that combines performance with respect to both traveled distance and energy consumption. However, it may be beneficial to analyze them separately in order to understand the limiting factors and to observe what can be the best compromise between them. The following experiment also allows us to qualitatively characterize the gaits of our structures and to observe possible transitions between different modes.

For this purpose, several optimizations have been performed by constraining the power and forcing their saturation to different values. In this way, one can expect to observe what is the maximum distance an individual can reach for a given power. Since we work outside the boundaries of the desirable operating range of the original cost function, we have now increased the reference value *D_ref_* to 1,000 m in order to avoid a saturation effect due to the cost function itself.

**Figure 7:**
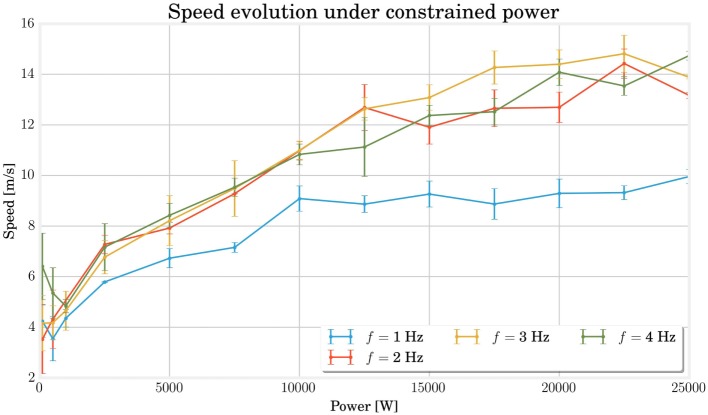
**This curves shows the evolution of the maximal speed reached for different constrained power at different frequencies**. The saturation effect for high powers demonstrates the physical limits of the structures, while the slight decrease for very low powers indicates a change in locomotion gaits associated with a different efficiency.

Figure 7 shows the evolution of the optimal speed as a function of a constrained power budget. The best individuals are in the upper left corner. As might be expected from the conclusions of the previous section, the 3-Hz frequency gives the best results. Concerning the shape of the curve, we can see that the maximum speed increases almost linearly until 15,000 W and starts saturating beyond that.

This saturation highlights the limits of our model. It helps to understand which factors such as the spring saturation, the ground friction, the air drag, or the geometry play a larger role in performance compared to the driving power. It also situates the previous experiments in the non-saturating range, which helps to appreciate their significance better.

Finally, for very low power, an energy increase does not seem to add any improvement and even the opposite happens for frequencies 1 and 4 Hz.

A visual observation of the locomotion is useful to give more insights about the possible gait transitions on this curve. For this purpose, we have produced a series of videos renditions of individual simulations provided in Supplementary Material. A qualitative analysis of those video shows that the most common gaits consist of displacing the whole structure along a wave movement (each node touches the ground a little after the previous one) or locomoting in two steps (the body touches the ground two times per period with a phase difference of 180°). Concerning the high power saturation, a video was made for each point of the 3-Hz curve. It shows that the most energy-consuming individuals present spring extension close to their saturation, which causes a loss of stability of the locomotion. In the same way, videos were produced in the low-power domain for the points on the 4-Hz curve. For the lowest power, a good two-step alternation of contacts between the body and the ground is observed, whereas the phase shifts between the different contacts with the ground are much less synchronous for the following individuals. The same results have been established each of the 5 times the experiment has been conducted. Progressively with increasing power, a two-step approach with robot–ground contacts phase-shifted by 180°comes up again.

In short, we can stress the role of the body design in locomotion through two principal observations: first, a saturation of the spring leading to a degraded operation in high power; second, a qualitative influence of the optimal gait on the performances for a given morphology and power consumption.

## 3 Closed-Loop Control

### 3.1 Materials and Methods

The closed-loop control of the MSD network is performed through physical reservoir computing. In this setup, our goal is to reproduce the control signals at a time step *t_k_* using the physical states of the network at times *t_k_*_−1−_*_n_* … *t_k_*_−1_ only. This is performed by training the weights of a linear combination (the readout).

#### 3.1.1 Setup

**Figure 8:**
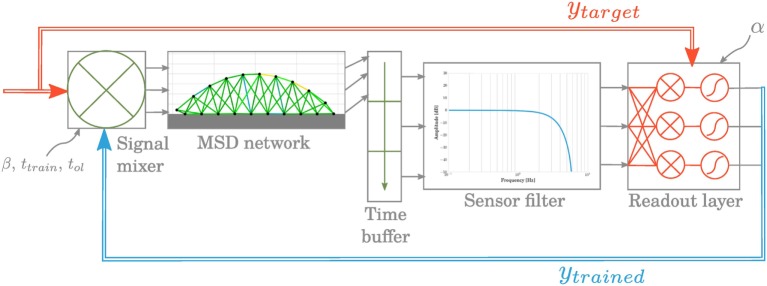
**The principal components in the closed-loop learning pipeline consist in a readout layer whose weight matrix is trained at each time step and a signal mixer that gradually integrates the feedback in the actuation signal**.

The closed-loop system is composed of different elements represented at Figure 8:

- The MSD structure that can be perceived as a physical reservoir because of its dynamics and high complexity. For each time step *t_k_*, the system’s current state is evaluated using the acceleration vectors **a**[*k*], **a**[*k −* 1], and **a**[*k −* 2], which comprise both X and Y components of all the nodes. The choice of acceleration, instead of, e.g., speed, is based on the work of Caluwaerts et al. (2013). Trials using integrated quantities such as position or speed instead have also been evaluated but added a drifting error during training. Also, based on the same work, we choose a buffer size of 3 time steps. In our experiments, smaller values led to deteriorated results but larger ones did not show any significant improvements.
- A sensor filter, whose principal role is to model the physical limitations in acceleration sensing. It is composed of an amplitude threshold followed by a low-pass filter. The cutoff frequency at 6 Hz has been chosen very low to eliminate possible oscillations due to our numerical integration method while keeping the locomotion fundamental frequency and its first-order harmonics. At the output of the filter, a vector **x**[*k*] is sent to the next element.
- A readout layer, which computes the actuation signals for the next time step based on the current and previous states of the MSD:
  (11) $$\text{y}\left[k+1\right]={\text{W}}_{\text{out}}^{T}\;.\;\text{x}\left[k\right].$$
  To learn the weights of the output matrix **W***_out_*, we use the FORCE learning method as in Sussillo and Abbott (2009), whose equations are the following:
  (12) $$\begin{array}{l} \text{e}\left[k+1\right]=f_{\text{sigmoid}}\left({\text{W}}_{\text{out}}^{T}\left[k\right]\;.\;\text{x}\left[k+1\right]\right)-{\text{y}}_{\text{target}}\left[k+1\right] \end{array}$$
  (13) $$\begin{array}{l} \text{P}\left[k+1\right]=\text{P}\left[k\right]-\frac{\text{P}\left[k\right]\;.\;\text{x}\left[k+1\right]\;.\;{\text{x}}^{T}\left[k+1\right]\;.\;\text{P}\left[k\right]}{1+\text{x}{\left[k+1\right]}^{T}\;.\;\text{P}\left[k\right]\;.\;\text{x}\left[k+1\right]} \end{array}$$
  (14) $$\begin{array}{l} {\text{W}}_{\text{out}}\left[k+1\right]={\text{W}}_{\text{out}}\left[k\right]-\text{P}\left[k+1\right]\;.\;\text{x}\left[k\right]\;.\;\text{e}{\left[k+1\right]}^{T} \end{array}$$
  (15) $$\begin{array}{l} {\text{y}}_{\text{trained}}\left[k+1\right]=f_{\text{sigmoid}}\left({\text{W}}_{\text{out}}^{T}\left[k+1\right]\;.\;\text{x}\left[k+1\right]\right), \end{array}$$
  where the estimate of the inverse of the correlation matrix **P** is initialized to **I**/α. The sigmoid function added ahead of the readout adds non-linearity in the control signal by saturating for too high values.
- A signal mixer to avoid a brutal transition from open-loop to closed-loop control. Its role is to incorporate gradually the readout output contribution to the target signal. It is defined by three parameters: the open-loop time *t*_ol_ when the MSD network is run in open-loop mode only; the training time *t*_train_ in which the contribution of closed-loop signal increases linearly and the percentage β of feedback in the full control signal before switching to closed-loop mode only.

#### 3.1.2 Parameter Tuning

**Figure 9:**
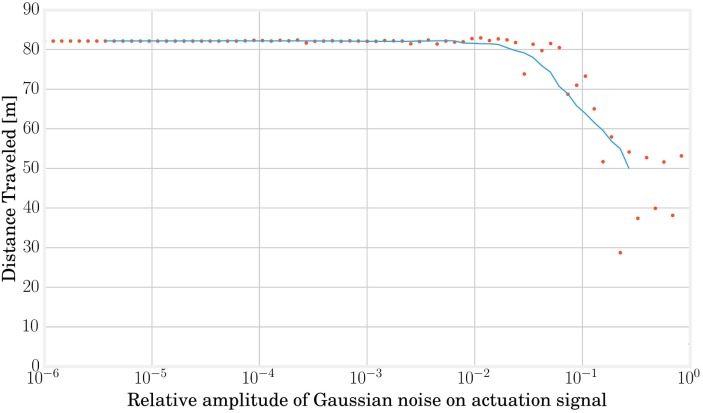
**Adding some noise on the actuation signal in open loop can give a hint about the maximum error we can accept on the trained signal in closed loop without damaging the locomotion stability and is helpful to determine the regularization parameter**. In this graph, each red point represents a simulation and the blue line shows the average evolution. Performances start decreasing from a relative Gaussian noise of 0.01.

The α parameter of the FORCE learning algorithm plays the role of a regularization variable in the process of learning the **W**_out_ matrix. It must be selected in order to avoid an overfitting that would reduce robustness to undesired forces on the MSD structure but also to ensure a trained signal sufficiently close to the target. This is a major issue since a signal **y**_trained_ with too much noise can easily cause a divergence in the locomotion limit cycle. Tests on signal noise robustness as presented in Figure 9 allowed to estimate a value of α = 0.01 as a good compromise.

**Figure 10:**
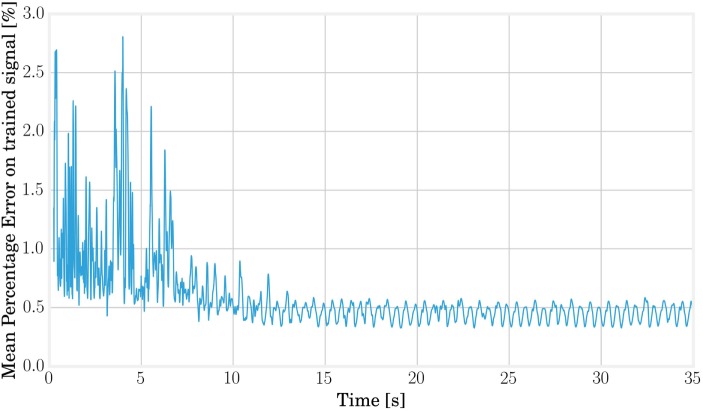
**The learning error can be used to estimate the required training time and the maximal rate at which the loop should be closed**. From this graph, we can deduce that 12 s of simulation is sufficient to consider the convergence of the readout weights.

The open-loop training and running times can be estimated by analyzing the convergence error of the FORCE algorithm (see Figure 10) and are fixed to 12 s of open-loop learning followed by 38 s where the feedback signal is gradually added to the target signal to reach a value of β = 95% before closing the loop. Stopping the training before the actuation signal reaches 100% of feedback avoids convergence to a steady state as discussed in Caluwaerts et al. (2013).

### 3.2 Results

**Figure 11:**
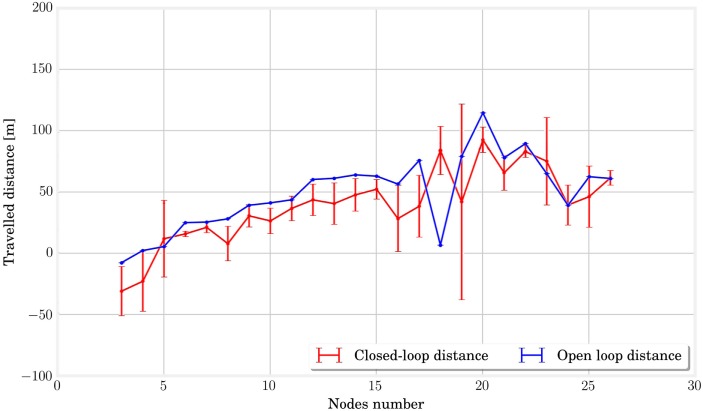
**In this picture, we plot the traveled distances for the last 10 s of simulation in open loop in blue and closed loop in red**. There is no crucial difference between the two curves, which seems to indicate that the performances in closed loop are close to the one in open loop for all structures.

In order to determine the contribution of the system size in the process of learning its own locomotion gaits, we simulated MSD networks with different numbers of nodes and evaluated the distances traveled over the last 10 s in closed loop. The same simulation was carried out in open loop to provide a reference. The results of these simulations are presented in Figure 11. At first sight, it appears that the learning algorithm with its configuration can achieve performances of the same order of magnitude in open and closed loops for the structures between three and twenty-six nodes analyzed in this simulation. However, it is worth noting that MSD with less than 6 nodes already provided non-significant results in open loop.

**Figure 12:**
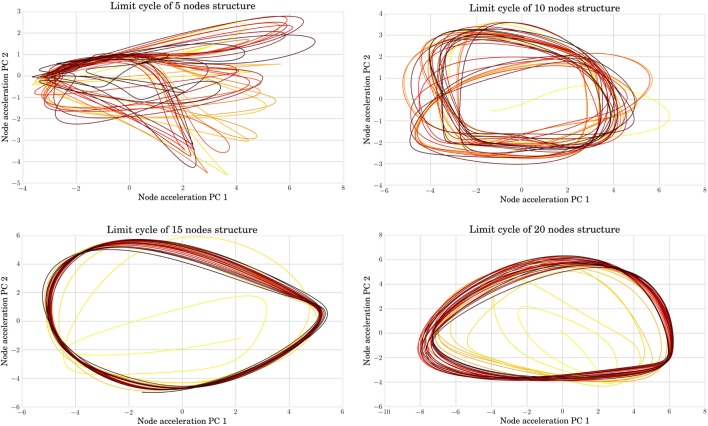
**From left to right and top to bottom, the limit cycle during FORCE training are represented for structures with, respectively, 5, 10, 15, and 20 nodes**. The color ranges from yellow for the initial seconds of the simulation (which point out the transient effect) to black in the end of the simulation. When the node number is too low, the trained signal can diverge from its basin of attraction.

**Figure 13:**
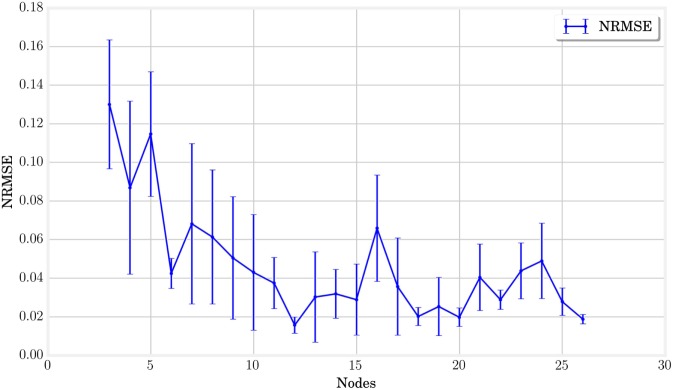
**The normalized root mean square error between the trained and target signals is represented for each node number**. It indicates that the learning tends to produce better more accurate results with an increasing number of nodes.

Alternatively, the study of limit cycles gives an indication of the stability of closed-loop control. In Figure 12, we represented the temporal evolution of the internal states **x***_k_* in a 2 coordinate space obtained by PCA. Larger structures lead to smoother limit cycles in closed loop. The limit cycles even diverge from their basin of attraction for very small MSD networks. A simple interpretation is that more nodes lead to more cycles in the physical reservoir, which provides more robust trajectories in the principal components reference. This hypothesis is corroborated by analyzing the quality of the generated actuation signals. This can be quantified by plotting the Normalized Root Mean Square Error, as shown in Figure 13, which decreases with the number of nodes.

In conclusion, the morphology of MSD bodies has the capability to compute at each time step the next value on the parametric trajectories found in open-loop optimization with a sufficient accuracy for locomotion task. The computation and memory that was previously embedded in an external controller can be fully distributed in the structure and the readout layer. The size and number of sensor measurements on the structure have a positive effect on the accuracy and stability of the feedback signal.

## 4 Discussion

In this article, we have tried to study systematically the influence of high-level design choices on the performance of MSD systems. Because of their analytical simplicity and their modularity, those body structures seem indeed adapted to conduct studies on the morphological contribution in the process of locomotion control. This research was divided into two main parts. On the one hand, an open-loop study focused on the benefits of body size to efficiency and stability. A similar analysis was also performed on locomotion frequency and helped to draw conclusions about how compliance can be chosen to increase optimal performance. On the other hand, we aimed at demonstrating the key role of morphology to generate control signals in a completely closed operation mode.

The different trials undertaken in open loop indicated the importance of the structure size to ensure optimal performance in terms of distance traveled and gait stability. Concerning compliance, its relation to the fundamental frequency of locomotion was used to demonstrate a link with the efficiency and to provide a specific suggestion in the design of optimal MSD systems. It has been noted that the frequency response of the different MSD networks shows a bell shape, displaying a degraded score for too high or too low frequencies and that the stability at high frequencies is better for larger structures. Finally, the behavior at different power values has highlighted the limits of the design in reaching high speeds, and a qualitative study has shown the effect of the gait evolution in this phenomenon.

In closed loop, the ability of MSD structures to generate their control signals on the basis of a single, fully connected layer of neurons has been attested. An increase in the size or the number of sensor signals induced a positive influence with regard to the limit cycle stability and the accuracy of the signals generated by the algorithm.

In future work, the main improvement should focus on increasing noise robustness and adaptability on different terrains and facing various obstacles. In this way, the goal is to provide a simple and generic locomotion primitive for complex structures, which learns how to perform actuator synchronization by harvesting the mechanical feedback while taking higher level control inputs such as the locomotion frequency. On the other hand, it would be interesting to generalize our conclusions to both real robots and biologically inspired dynamical models such as quadrupeds and bipeds.

## Author Contributions

The experiments were conceived by GU, BC, FW, JDegrave, and JDambre and designed by GU and BC. The data were analyzed by GU with help of FW, JDegrave, and JDambre. The manuscript was mostly written by GU, with comments and corrections from FW and JDambre.

## Conflict of Interest Statement

The authors declare that the research was conducted in the absence of any commercial or financial relationships that could be construed as a potential conflict of interest.

## Footnotes

^1^https://github.com/Gabs48/SpringMassNetworks.

## Supplementary Material

Video S1 **This video presents several simulation renditions**. The different locomotion processes displayed are learned through optimization in open-loop control. It aims at providing the reader a qualitative understanding of the different gait types obtained when constraining the dissipated power at different levels. A discussion on that matter is given in Section 2.2.3 of the related article.
